# Prevalence estimates of substandard drugs in Mongolia using a random sample survey

**DOI:** 10.1186/2193-1801-3-709

**Published:** 2014-12-02

**Authors:** Daariimaa Khurelbat, Gereltuya Dorj, Enkhtuul Bayarsaikhan, Munkhdelger Chimedsuren, Tsetsegmaa Sanjjav, Takeshi Morimoto, Michael Morley, Katharine Morley

**Affiliations:** School of Pharmacy and Biomedicine, Mongolian National University of Medical Sciences, Ulaanbaatar, Mongolia; School of Pharmacy Curtin University of Technology, Perth, Western Australia; Ministry of Health, Division of Pharmaceuticals and Medical Devices, Ulaanbaatar, Mongolia; Ministry of Health, Fourth Health Sector Development Project, Ulaanbaatar, Mongolia; Division of General Internal Medicine, Hyogo College of Medicine, Nishinomiya, Japan; Department of Ophthalmology, Harvard University, Boston, MA USA; Department of Medicine, Harvard University, Boston, MA USA

**Keywords:** Medication quality, Substandard, Falsified, Patient safety, Asia, Developing countries

## Abstract

To determine the prevalence of substandard drugs in urban (Ulaanbaatar) and rural (selected provinces) areas of Mongolia, samples of 9 common, therapeutically important drugs were collected from randomly selected drug outlets in Ulaanbaatar and 4 rural provinces by “mystery shoppers”. Samples were analyzed by visual inspection, registration status, and biochemical analysis. Samples failing to meet all Pharmacopeia quality tests were considered substandard.

In the rural provinces, 69 out of 388 samples were substandard, giving an estimated prevalence of substandard drugs of 17.8% (95% CI: 14.1-22.0). There were 85 unregistered samples, giving a prevalence estimate of unregistered drugs of 21.9%. (95% CI: 17.9-26.3). In the urban Ulaanbaatar districts, 112 out of 848 samples were substandard, giving an estimated prevalence of substandard drugs of 13.2% (95% CI: 11.0-15.7). There were 150 unregistered samples, giving a prevalence estimate of unregistered drugs of 17.7% (95% CI: 15.2-20.4).

In the rural provinces, 35 out of 85 (41.2%) unregistered samples were substandard; whereas 34 out of 303 (11.2%) registered samples were substandard. (p < 0.0001) In the urban districts, 18 out of 150 (12.0%) unregistered samples were substandard, whereas 94 out of 698 registered were substandard. (13.5%) (p = 0.6).

The prevalence of substandard and unregistered drugs is higher in rural provinces. There is a significant association between substandard and unregistered drugs in the provinces but not in the urban districts. The underlying causes for substandard drugs need to be further investigated in order to help formulate strategies to improve pharmacovigilance and the drug supply quality in Mongolia.

## Background

Poor quality drugs have been increasingly recognized as a global public health threat because they have the potential to result in inadequate treatment, cause adverse effects from toxic ingredients, and promote drug resistance. The nomenclature of the categories of poor quality medications can be confusing. The World Health Organization recently chose to group all categories together as “SSFFC”: substandard, spurious, falsely-labeled, falsified, and counterfeit. Revision of these categories as: “substandard” - drugs that for unintentional reasons do not meet the legally required quality specifications of a country’s regulators, “unregistered” - drugs that do not have the legally required marketing authorization from the country’s regulators, and “falsified” - drugs that are unlawful, and violate the regulators quality specifications, with criminal intent was subsequently suggested (Attaran et al. 2012). Fernandez, et al. raise the issue that a genuine drug found to have an insufficient amount of an active ingredient could be substandard or degraded (Fernandez et al. 2011), indicating poor quality drugs can result from issues in production or external factors such as environmental conditions, impacting quality after distribution.

The true extent of the problem is difficult to ascertain. Reasons for this include the difficulty and expense in performing a methodologically sound study, reluctance of governments to disclose information and the fact that many of the effects on patients are difficult to detect and hidden in other public health statistics (Cockburn et al. 2005). In his 2010 article, Newton states there is an urgent need for data of sufficient sample size, with random sampling design to reliably estimate the prevalence of poor quality medicines (Newton, et al. 2010) Literature reviews of prevalence studies on falsified/substandard drugs report that the percentage of substandard drugs in various Asian and African countries range from 8-46% (Caudron et al. 2008), and the median prevalence of substandard/falsified medicines was 28.5% (range 11–48%) (Almuzaini et al. 2013). The World Health Organization (WHO) conducted a survey on the quality of selected anti-malarial medication in 6 subSaharan African countries, which found that 28.5% of the samples failed to meet testing requirements, with 11.6% having extreme deviations, and therefore likely to have negative health implications (Sabartova et al. 2011a). Another WHO survey was conducted on the quality of anti-tuberculosis medications in Russia, and found 11.3% of the samples failed to meet study specifications, with 1.0% having extreme deviations (Sabartova et al. 2011b). In 1999, WHO conducted a survey of drug quality in Myanmar and Vietnam, and found that 16% of the samples did not meet all specifications of testing (Wondemagegnehu 1999).

Between 2004–2006 the pharmaceutical procurement system in Mongolia underwent decentralization, and is now 100% privatized. In the current system, the Division of Pharmaceutical and Medical Devices, Mongolian Ministry of Health (MoH) is responsible for the policy, planning and regulatory affairs in providing pharmaceutical care in Mongolia. The special licenses for manufacturing, importing, purchasing pharmaceuticals and medical devices are granted by the Special Permission Committee of the MoH. Drugs are distributed through drug wholesalers and retail drug outlets (community pharmacies and revolving drug funds (RDF)). Wholesalers can import and procure drugs with an approval and special permission from the Mongolian Minister of Health. In 2011, there were 158 registered drug wholesaling companies and 42 local drug manufacturing companies, some of which act as both wholesalers and retailers. Approximately 85% of all drugs are imported from other countries, primarily Russia and India, followed by Germany, Slovenia and China.

Poor quality drugs have been a concern in Mongolia, supported by the findings from a 2006 study on unregistered, falsified and substandard drugs (Mongolia Ministry of Health 2006). Using convenience sampling methods, 225 samples were collected from 40 drug outlets around the country, 55 of which were felt to be “suspicious” and were sent for further testing. Sixteen of these were felt to be “inconsistent” and 8 were possibly counterfeit. A 2008 study by Tsetsegmaa found that 11 of the 16 medications reported in the surveillance were substandard (Tsetsegmaa 2008). In a 2009 report, lack of knowledge about the effectiveness of drug quality monitoring in Mongolia was reported as a gap that should be a priority for further investigation (Abdelkrim 2009).

This research study was undertaken to address these concerns, and provide data of good methodological quality to accurately determine the prevalence of substandard drugs in the rural and urban areas of Mongolia after the decentralization and privatization of the Mongolian pharmaceutical system. This information will be of value to Mongolian policy makers, public health officials and pharmaceutical practitioners to reliably determine the extent of the problem, and then can serve as a valid comparison for future studies to evaluate interventions to improve the drug supply quality. It will also help guide further research to better understand the health impact of poor quality medications in Mongolia.

## Methods

### Site selection

Mongolia is a landlocked country in north central Asia, with 21 rural provinces, plus 1 municipality, the capital city of Ulaanbaatar where over 60% of the population lives. Because the conditions in rural provinces vary greatly from the urban area of Ulaanbaatar, samples were collected, analyzed, and reported independently. Samples for this study were collected from 4 districts in Ulaanbaatar (Chingeltei, Khan-Uul, Bayanzurkh, and Songinokhair) and 4 rural provinces (Bayan-Uglii, Dornogobi, Selenge, and Umnugobi) representing the main geographic regions of the country. Samples were obtained from the different types of drug outlets in the provinces: Revolving Drug Fund (RDF- a government outlet), retail pharmacy outlets, and wholesalers. In Ulaanbaatar districts, samples were only obtained from retail pharmacy outlets and wholesalers, as RDF outlets are only present in the provinces. Samples from unofficial drug outlets and the informal market were not included in this study.

Medications included in the study were selected based on high therapeutic importance and utilization based on discussions with local experts from Schools of Pharmacy, Public Health, and Mongolian National University of Medical Sciences. They are all on the Essential Drug List and available with or without a prescription. All samples were tablets or capsules and include antimicrobials (ampicillin, amoxicillin, co-trimoxazole, metronidazole, doxycycline, nystatin), analgesics (paracetamol and ibuprofen), and bromhexin, a commonly used medication for respiratory illness (Table 1).

**Table 1:** **Drugs in study population**

Name of drug	Dosage form	Pharmacopeia reference
Metronidazole	250 mg/tab	Mongolian National Pharmacopeia 2011
		Pharmacopeia of the People’s Republic of China 2005. Vol. II,
Nystatin	500000 ID/tab	British Pharmacopeia 2001. Vol.2
Ibuprofen	400 mg/tab	Mongolian National Pharmacopeia 2011
		Pharmacopeia of the People’s Republic of China 2005. Vol. II,
Co-trimoxazole	480 mg/tab	Mongolian National Standard-MNS 6149-2010
Amoxicillin	500 mg/cap	Mongolian National Pharmacopeia 2011
Paracetamol	500 mg/tab	Mongolian National Pharmacopeia 2011
		Pharmacopeia of the People’s Republic of China 2005. Vol. II,
Ampicillin	500 mg/cap	British Pharmacopeia 2001. Vol. 2
		Mongolian National Pharmacopeia 2011
		USP 23
Bromhexin	8 mg/tab	Mongolian National Pharmacopeia 2011
		Pharmacopeia of the People’s Republic of China 2005. Vol. II,
Doxycycline	100 mg/cap	Mongolian National Standard-MNS 5776–2007
		Pharmacopeia of the People’s Republic of China 2005. Vol. II,

### Sample size calculation

Prevalence studies from other countries indicate a wide range of substandard drugs, 8-46% (Caudron et al. 2008), and 11–48% (Almuzaini et al. 2013). Based on this information and the previous studies of falsified/substandard drugs in Mongolia, we targeted our sample size to detect at least a 5% prevalence (alpha of 0.05 and beta of 0.9). This calculation was 134 samples for each drug (1206 for all drug types combined) distributed among the provinces or districts. In order to detect a 10% prevalence, the sample size needed was 67 (603 combined) and 15% prevalence was 38 samples (342 combined).

### Sampling techniques

The sampling strategy included weighting the sample size by population and the number of the types of drug outlets in the province or district. Drug outlets to be sampled were selected randomly.

A sample was defined as 100 dosage units (tablet or capsule) of a given drug of the same lot number purchased in blister packs of 10 dosage units.

Samples were collected from the 4 provinces between May 2012 and September 2012 and from the 4 Ulaanbaatar districts between July 2012 and March 2013 by “mystery shoppers”. These were trained field workers, who presented themselves as local customers, and followed the study protocol for obtaining drug samples based on recommended sampling techniques (Newton et al. 2009). If they were unable to purchase the necessary quantity for a complete sample from one batch or lot, this was noted and attempts were made to purchase it from another randomly selected outlet of the same type. Collected samples were placed in a box, then transported to and stored in lockers at the School of Pharmacy, Mongolian National University of Medical Sciences. The transport box and lockers met the temperature and humidity requires of the WHO Guidelines for the Sampling of Pharmaceutical Products, and were accessible only by the main study investigator.

### Sample analysis

Sample analysis for each sample consisted of visual inspection of the packaging and labeling, and determination of registration status, expiration date, country of manufacture, biochemical analysis, and company of manufacture. An online database developed by the Ministry of Health in Mongolia (Licemed) and archive documents from the registration of drugs were used to complete the visual inspection. The database includes information such as size, color, labeling and numbers of the packages and labeling. In addition, the WHO guideline for the Development of Measures to Combat Counterfeit Drugs was used. (World Health Organization 1999) A sample was considered suspicious if the package and labeling was not consistent with registered information for that drug and manufacturer. Samples with suspicious packaging and labeling were sent to the manufacturers for confirmation. If the manufacturer confirmed that it was their product, the sample was considered acceptable.

The registration status of all samples was determined by visual inspection of the packaging, and then confirmed using the drug registration archives at the Mongolian Ministry of Health. Registration was not considered a requirement for determining whether or not a sample was substandard.

Drug samples underwent biochemical analysis by 1 of 3 laboratories in Mongolia: Drug and Bio-preparation Central Laboratory of Specialized Professional Inspection Agency (SPIA); Drug Control Laboratory, School of Pharmacy, Mongolian National University of Medical Sciences; and the Drug Testing Laboratory “Monos Group”. These laboratories are accredited by the Standardization and Technical Regulatory Office of the Centre for Standardization and Measurement in Mongolia, which is responsible for the technical standards in local production and quality control. The Pharmacopoeias were chosen according the country of origin of the sample or specification requirements of the manufacturer (Table 1). (British Pharmacopoeia 2001, Mongolian Pharmacopeia 2011, Pharmacopeia of the People’s Republic of China 2005). These requirements vary by drug, and include 8–11 of the following tests: appearance, assay, disintegration, dissolution, hardness, identification, irradiance absorption, water, friability, weight average and weight variation (Table 2). The qualitative analysis included: 1). visual inspection of package and labeling, 2). characteristics of the sample (appearance, odor, color dosage form), 3). uniformity of weight, disintegration, and dissolution, 4). identification of components by chemical reaction, and thin layer chromatography, spectrum analysis on UV spectrophotometer and IR spectrophotometer. Quantitative analysis included assay of active compounds by spectrophotometric, titrometric and chromatographic methods. A sample was considered to be substandard if it failed to pass all required tests for the drug required by the article requirements in the Pharmacopeia used, that is, if the sample failed one or more of the required tests it was considered substandard.

**Table 2:** **Sample analysis definitions**

Test	Definition
Appearance	Clean, smooth surface and uniform color of tablet or capsule
Friction and substantial	Tablet crushing strength
Weight average	Average weight of 20 tablets
Weight variation	Difference between the weight of the content of each solid form and the average weight of solid forms
Disintegration	Disintegration or disbursement of solid preparations into fragments or particles in a liquid medium
Dissolution	Rate and degree of dissolution of active ingredients in liquid medium
Content uniformity	Contents of single ingredient solid preparations
Water (Loss on drying)	Determine water loss on drying
Identification	Verify identity by visual inspection
Irradiance absorption	Absorbance in the ultraviolet region
Assay	Determine content of active ingredients

### Ethics approval

Ethics approval was obtained from the World Health Organization Ethics Review Committee and the Medical Ethics Committee of the Ministry of Health, Mongolia.

### Statistical analyses

Measurements were presented as numbers and percentages with 95% confidence intervals (CIs), and were compared with the chi-square test or Fisher’s exact test. P values <0.05 were regarded as statistically significant.

## Results

### Description of sample and analysis results

#### Sample description

The number of samples collected for this study was 388 from the rural provinces and 848 from the urban districts of Ulaanbaatar. The distribution of the samples based on location by drug outlet type is presented in Table 3, and location by drug in Table 4.

**Table 3:** **Number of samples by location and drug outlet type**

	Wholesale		Retail		RDF*		Total	
	N	%	N	%	N	%	N	%
**Rural provinces**								
Bayan-Ulgii	15	3.9	77	19.8	34	8.8	126	32.5
Dornogobi	14	3.6	30	7.7	36	9.3	80	20.6
Selenge	12	3.1	52	13.4	58	14.9	122	31.4
Umnugobi	10	2.6	27	7.0	23	5.9	60	15.5
**All provinces**	**51**	**13.1**	**186**	**47.9**	**151**	**38.9**	**388**	**100**
**Urban districts**								
Bayanzurkh	41	4.8	248	29.2	NA	NA	289	34.1
Chingeltei	50	5.9	111	13.1	NA	NA	161	19.0
Khan-Uul	32	3.8	97	11.4	NA	NA	129	15.2
Songinokhairkhan	26	3.1	243	28.7	NA	NA	269	31.7
**All districts**	**149**	**17.6**	**699**	**82.4**	**NA**	**NA**	**848**	**100**

*RDF: Revolving Drug Fund (government outlet).

**Table 4:** **Number of samples by drug and location**

	Amoxicillin		Ampicillin		Bromhexin		Co-trimoxazole		Doxycycline		Ibuprofen		Metronidazole		Nystatin		Paracetamol		Total	
**Rural province**	**N**	**%**	**N**	**%**	**N**	**%**	**N**	**%**	**N**	**%**	**N**	**%**	**N**	**%**	**N**	**%**	**N**	**%**	**N**	**%**
Bayan-Ulgii	17	4.4	14	3.6	13	3.4	17	4.4	10	2.6	13	3.4	14	3.6	15	3.9	13	3.4	126	32.5
Dornogobi	11	2.8	8	2.1	8	2.1	10	2.6	10	2.6	8	2.1	8	2.1	9	2.3	8	2.1	80	20.6
Selenge	12	3.1	13	3.4	14	3.6	14	3.6	14	3.6	13	3.4	13	3.4	18	4.6	11	2.8	122	31.4
Umnugobi	6	1.5	5	1.3	6	1.5	6	1.5	9	2.3	9	2.3	6	1.5	7	1.8	6	1.5	60	15.5
**All provinces**	**46**	**12**	**40**	**10**	**41**	**11**	**47**	**12**	**43**	**11**	**43**	**11**	**41**	**11**	**49**	**13**	**38**	**10**	**388**	**100**
**Urban district**																				
Bayanzurkh	33	3.9	37	4.4	41	4.8	30	3.5	27	3.2	31	3.7	37	4.4	31	3.7	22	2.6	289	34.1
Chingeltei	24	2.8	15	1.8	13	1.5	18	2.1	17	2.0	20	2.4	21	2.5	19	2.2	14	1.7	161	19.0
Khan-Uul	14	1.7	15	1.8	19	2.2	14	1.7	11	1.3	12	1.4	16	1.9	16	1.9	12	1.4	129	15.2
Songinokhairkhan	30	3.5	27	3.2	33	3.9	33	3.9	28	3.3	32	3.8	35	4.1	34	4.0	17	2	269	31.7
**All districts**	**101**	**11.9**	**94**	**11.1**	**106**	**12.5**	**95**	**11.2**	**83**	**9.8**	**95**	**11.2**	**109**	**12.9**	**100**	**11.8**	**65**	**7.7**	**848**	**100**

#### Sample inspection

Out of 388 samples from the rural provinces, only 3 were found to be past expiration date. There were 4 others that expired within the data collection period of May to August 2012, so may have recently expired. Out of 848 samples from the Ulaanbaatar districts, none were found to be past expiration date.

On initial inspection, 22 drug samples from the rural provinces and urban districts combined were found to have variation in the packaging and labeling of the drugs when compared with the products registered in Mongolia. Upon review by the manufacturer, all 22 were found to be acceptable or meeting standards due to packaging updates.

#### Biochemical sample analysis

Failure to pass the assay test (e.g. amount of required ingredients fell outside range of Pharmacopeia standards) was the most common reason that a sample was found to be substandard. Failure to pass this test indicates that the sample did not meet the threshold requirements regarding amount of drug present and does not give any information about the degree or direction of deviation from the required standard (Table 5). In the provincial group, 51 out of 388 (13.4%, 95% CI: 9.9-16.9) samples failed the assay test. The other common reasons were weight variation and weight average. There were a few samples failing tests for dissolution, disintegration and friction (Table 6). In the Ulaanbaatar district samples, 55 out of 848 (6.6%, 95% CI: 4.9- 8.4 failed the assay test (Table 5). The other common reasons were disintegration and dissolution. There were a few samples that failed the following tests weight variation, weight average, and friction (Table 7).

**Table 5:** **Number of samples failing assay by location**

	Rural			Urban		
	N	%	95% CI*	N	%	95% CI*
**Failed assay**	51	13.1	9.9, 16.9	55	6.5	4.9, 8.4
**Passed assay**	337	86.9	83.1, 90.1	793	93.5	91.6, 95.1
**Total**	**388**	**100**		**848**	**100**	

*CI: confidence interval

**Table 6:** **Sample analysis for drugs by acceptability from rural provinces**

	Amoxicillin		Ampicillin		Bromhexin		Co-trimoxazole		Doxycycline		Ibuprofen		Metronidazole		Nystatin		Paracetamol		Total	
	#	%	#	%	#	%	#	%	#	%	#	%	#	%	#	%	#	%	#	%
**Not acceptable**																				
Assay	2	1%	0	0%	0	0%	6	2%	4	1%	11	4%	17	6%	7	3%	4	2%	51	2%
Disintegration	0	0%	0	0%	0	0%	1	0%	0	0%	5	2%	1	0%	0	0%	0	0%	7	0%
Dissolution	1	0%	0	0%	0	0%	0	0%	0	0%	5	2%	2	1%	0	0%	3	1%	11	0%
Friction	0	0%	0	0%	0	0%	5	1%	0	0%	0	0%	0	0%	0	0%	0	0%	5	0%
Wt average	0	0%	0	0%	0	0%	1	0%	0	0%	0	0%	15	5%	0	0%	0	0%	16	1%
Wt variation	1	0%	0	0%	0	0%	3	1%	1	0%	5	2%	12	4%	0	0%	5	2%	27	1%
**Not acceptable total**	**4**	**1%**	**0**	**0%**	**0**	**0%**	**16**	**5%**	**5**	**2%**	**26**	**9%**	**47**	**16%**	**7**	**3%**	**12**	**5%**	**117**	**4%**
**Acceptable**																				
Appearance	46	14%	40	14%	41	17%	47	14%	43	14%	43	14%	41	14%	49	17%	38	14%	388	15%
Assay	44	13%	40	14%	41	17%	41	12%	39	13%	32	11%	24	8%	42	14%	34	13%	337	13%
Disintegration	46	14%	40	14%	41	17%	46	14%	43	14%	38	13%	40	14%	49	17%	38	14%	381	14%
Dissolution	45	14%	40	14%	2	1%	5	1%	43	14%	38	13%	39	14%	0	0%	35	13%	247	9%
Friction	0	0%	0	0%	0	0%	40	12%	0	0%	0	0%	0	0%	0	0%	0	0%	40	2%
Identification	46	14%	40	14%	41	17%	47	14%	43	14%	43	14%	41	14%	49	17%	38	14%	388	15%
Irradiance absorption	0	0%	0	0%	0	0%	0	0%	5	2%	0	0%	0	0%	0	0%	0	0%	5	0%
Substantial	0	0%	0	0%	0	0%	2	1%	0	0%	0	0%	0	0%	0	0%	0	0%	2	0%
Water	4	1%	0	0%	0	0%	0	0%	0	0%	0	0%	0	0%	0	0%	0	0%	4	0%
Wt average	46	14%	40	14%	41	17%	46	14%	43	14%	43	14%	26	9%	49	17%	38	14%	372	14%
Wt variation	45	14%	40	14%	41	17%	44	13%	42	14%	38	13%	29	10%	49	17%	33	12%	361	14%
**Acceptable total**	**322**	**99%**	**280**	**100%**	**248**	**100%**	**318**	**95%**	**301**	**98%**	**275**	**91%**	**240**	**84%**	**287**	**97%**	**254**	**95%**	**2525**	**96%**
**Grand total**	**326**	**100%**	**280**	**100%**	**248**	**100%**	**334**	**100%**	**306**	**100%**	**301**	**100%**	**287**	**100%**	**294**	**100%**	**266**	**100%**	**2642**	**100%**

**Table 7:** **Sample analysis for drugs by acceptability from urban districts**

	Amoxicillin		Ampicillin		Bromhexin		Co-trimoxazole		Doxycycline		Ibuprofen		Metronidazole		Nystatin		Paracetamol		Total	
	#	%	#	%	#	%	#	%	#	%	#	%	#	%	#	%	#	%	# %	
**Not Acceptable**																				
Assay	9	1%	0	0%	0	0%	0	0%	8	1%	8	1%	6	1%	23	4%	1	0%	55	1%
Disintegration	0	0%	0	0%	0	0%	2	0%	0	0%	36	5%	0	0%	0	0%	6	1%	44	1%
Dissolution	0	0%	0	0%	0	0%	1	0%	1	0%	8	1%	5	1%	0	0%	5	1%	20	0%
Friction	0	0%	0	0%	0	0%	0	0%	0	0%	1	0%	0	0%	0	0%	0	0%	1	0%
Wt Average	0	0%	0	0%	0	0%	0	0%	0	0%	0	0%	3	0%	0	0%	0	0%	3	0%
Wt Variation	2	0%	0	0%	0	0%	0	0%	7	1%	2	0%	3	0%	0	0%	1	0%	15	0%
**Not Acceptable Total**	11	2%	0	0%	0	0%	3	0%	16	3%	55	8%	17	2%	23	4%	13	3%	138	2%
**Acceptable** Appearance	101	14%	94	14%	106	14%	95	13%	83	14%	95	13%	109	14%	100	17%	65	14%	848	14%
Assay	92	13%	94	14%	106	14%	95	13%	75	13%	87	12%	103	13%	78	13%	64	14%	793	13%
Disintegration	101	14%	94	14%	106	14%	91	12%	83	14%	59	8%	109	14%	100	17%	59	13%	802	13%
Dissolution	101	14%	94	14%	2	0%	93	12%	82	14%	86	12%	101	13%	2	0%	58	13%	619	10%
Friction	1	0%	0	0%	104	14%	94	12%	1	0%	62	9%	3	0%	1	0%	0	0%	266	4%
Identification	101	14%	94	14%	106	14%	95	13%	83	14%	95	13%	109	14%	100	17%	65	14%	848	14%
Wt Average	101	14%	94	14%	106	14%	95	13%	83	14%	95	13%	106	14%	100	17%	65	14%	845	14%
Wt Variation	99	14%	94	14%	106	14%	95	13%	76	13%	93	13%	106	14%	100	17%	64	14%	833	14%
**Total Acceptable**	697	98%	658	100%	742	100%	753	100%	566	97%	672	92%	746	98%	581	96%	440	97%	5854	98%
**Grand Total**	**708**	**100%**	**658**	**100%**	**742**	**100%**	**756**	**100%**	**582**	**100%**	**727**	**100%**	**763**	**100%**	**603**	**100%**	**453**	**100%**	**5992**	**100%**

### Prevalence of substandard drugs

#### Rural provinces

Out of 388 samples collected from all 4 rural provinces, 69 were classified as substandard. This gives a substandard drug prevalence rate of 17.8% (95% CI: 14.1-22.0) in the rural provinces (Table 8).

**Table 8:** **Prevalence of substandard drug samples by location**

	Rural			Urban		
	N	%	95% CI*	N	%	95% CI*
**Substandard**	69	17.8	14.1, 22.0	112	13.2	11.0, 15.7
**Acceptable**	319	82.2	78.0, 85.9	736	86.8	84.3, 89.0
**Total**	**388**	**100**		**848**	**100**	

*CI: confidence interval.

#### Urban districts

Out of 848 samples collected from all 4 urban districts of Ulaanbaatar, 112 were classified as substandard. This gives a prevalence rate of 13.2% (95% CI: 11.0-15.7) substandard drugs in the urban districts of Ulaanbaatar (Table 8).

### Registration status

#### Rural provinces

Out of 388 samples collected from the 4 provinces, 85 were unregistered. This gives a prevalence estimate of unregistered drugs in the provinces of 21.9%. (95% CI: 18.0-26.3) (Table 9). Out of the 85 unregistered samples, 35 were substandard (41.2%), compared with 34 substandard samples out of the 303 registered samples (11.2%). This is a statistically significant difference (p < 0.0001) (Table 10).

**Table 9:** **Prevalence of unregistered drug samples by location**

	Rural			Urban		
	N	%	95% CI*	N	%	95% CI*
**Unregistered**	85	21.9	18.0, 26.3	150	17.7	15.2, 20.4
**Registered**	303	78.1	73.6,82.1	698	82.3	79.6, 84.8
**Total**	**388**	**100**		**848**	**100**	

*CI: confidence interval.

**Table 10:** **Substandard samples by location and registration status**

	Substandard		Acceptable		Total	
Rural provinces	N	%	N	%	N	% Substandard
Unregistered	35	9.0	50	12.9	85	41.2
Registered	34	8.8	269	69.3	303	11.2
**All provinces**	**69**	**17.8**	**319**	**82.2**	**388**	
**Urban districts**	**N**	**%**	**N**	**%**	**N**	**% Substandard**
Unregistered	18	2.1	132	15.6	150	12.0
Registered	94	11.1	604	71.2	698	13.5
**All districts**	**112**	**13.2**	**736**	**86.8**	**848**	

#### Districts of Ulaanbaatar

Out of 848 samples, collected from the 4 districts of Ulaanbaatar, 150 were unregistered. This gives a prevalence estimate of unregistered drugs in the Ulaanbaatar districts of 17.7% (95% CI: 15.2-20.4) (Table 9). Out of 150 unregistered samples, 18 were substandard (12.0%), compared with 94 substandard samples out of the 698 registered samples (13.5%). This difference is not statistically significant (p = 0.6) (Table 10).

## Discussion

Our results provide prevalence estimates for substandard drugs in Mongolia of 17.8% in the rural provinces and 13.2% in the urban districts of Ulaanbaatar, based on failure to meet the threshold quality standards established in the selected Pharmacopeia. While our study design does not allow us to directly compare these results from these 2 regions, it is interesting to note a modestly higher prevalence of substandard drugs in the rural sample. We also noted a significant association between substandard and unregistered drugs in the provinces, but not in the urban districts.

Our prevalence estimates of substandard drugs of 17.8% and 13.2% in Mongolia are in alignment with the range of 11-14% reported by Almuzaini et al. in their recent review of substandard and falsified medications in low and middle income countries in Asia and Africa (Almuzaini et al. 2013). Our prevalence estimates are lower than the median percentage of 28% reported in this review, however, this comparison is limited by the differences in methodology, sample size, inclusion criteria and drugs selected between the various studies reported and ours. The most common reason for a sample to be substandard was failure to pass assay test, which is consistent with the findings of other studies (Almuzaini et al. 2013). Failure to pass the assay test, along with failure to pass the disintegration and dissolution tests, the other most common reasons in our study, indicates that the bioavailability of the active ingredients was compromised. This can lead to ineffective treatment, and in the case of antibiotics, promote drug resistance. Of note, almost none of the samples were found to be post-expiration date, suggesting other factors are contributing to the degradation in drug quality. Further investigation into drug transport and storage conditions may help better understand this, especially given the extreme weather conditions found in Mongolia.

Another interesting finding of our study was the 21.9% prevalence of unregistered drugs in the provinces and 17.7% in the districts of Ulaanbaatar. This raises the importance of further investigation of the drug supply chain and evaluation of drug regulatory policies. Such initiatives could be undertaken at the national level and through collaborations with neighboring countries. We believe this may be an especially important step to improve the quality of the drug supply in the provinces where there was a statistically significant association between unregistered and substandard drug samples.

An adequate sample size is essential to obtaining valid results. Our sample size calculations indicated that we would need 342 samples for each region to detect a 15% prevalence. We achieved this in both the rural provinces (N = 388, 17.8% prevalence) and the urban districts (N = 848, 13.2% prevalence). However, there are some weaknesses in our study that could underestimate our prevalence estimates. These include the potential for drug outlet personnel to selectively provide drugs if they were suspicious about the reason for the purchase, and excluding drug samples from the unlicensed market, where the prevalence of substandard drugs has found to be significantly higher (Almuzaini et al. 2013). Another potential issue is that the biochemical analysis was performed at 3 different drug testing laboratories in Mongolia. Although they all used the same Pharmacoepeia standards, the possibility of variability in testing between facilities exists. In order to confirm the accuracy of the results, we had planned to send 10% of the samples to an outside lab for verification. Because of budgetary constraints, only 4 substandard samples (2.2%) were actually sent for testing at an outside reference laboratory (National Institute of Drug Quality Control of Vietnam, Hanoi, Vietnam). These 4 samples were all verified as correctly classified, but it is not a large enough number and did not include any acceptable samples, therefore we cannot claim to validate our findings by outside reference laboratory testing.

Another important limitation of our study is that it does not provide any details about the degree of variation from the threshold requirements of the Pharmacopeia quality standards. Our study also does not provide any information about the presence of harmful ingredients. Because of this, our ability to make any inferences about the potential clinical, safety, or economic impact of the substandard drugs in Mongolia is limited, but it does support the need for increased pharmacovigilance and review of drug regulatory policies. Further details of the biochemical analysis of the substandard samples, particularly the degree and direction of the deviation of the samples failing the assay, could provide additional valuable insight into the public health impact of poor drug quality.

## Conclusions

Our findings indicate that the presence of substandard drugs raise a genuine concern in both urban and rural areas of Mongolia. In addition, we found that unregistered drugs are common in both areas, with a significant association between substandard and unregistered drugs in the rural provinces. This highlights an important opportunity to improve the quality of the drug supply in Mongolia by reviewing and enforcing drug registration and inspection polices. Improving drug storage conditions and importation monitoring at borders are other interventions that can potentially improve drug supply quality, especially in rural provinces. Other areas for further investigation to better understand the quality of the drug supply in Mongolia would be to determine the degree of variation in the assay results for substandard drug samples, sampling the unlicensed market, and investigating the drug supply chain, especially in the provinces. Another important area for further study of the public health impact of substandard drugs is evaluating the patterns of antibiotic resistance and health outcomes for people living in areas with a high prevalence of substandard drugs.
